# Hormonal Status and Quality of Life of Women Treated for Infertility Before and During COVID-19 Pandemic in Poland

**DOI:** 10.3390/jcm14030721

**Published:** 2025-01-23

**Authors:** Kamila Wójtowicz, Justyna Kot, Marta Makara-Studzińska, Natalia Wdowiak, Michał Filip, Andrzej Wróbel, Jan Wróbel, Dorota Matuszyk, Melania Bojar, Joanna Bartosińska, Artur Wdowiak

**Affiliations:** 1 Medical Center Łańcut Gynecology and Obstetrics Department, 37-100 Łańcut, Poland; guccii@poczta.onet.pl; 2 Faculty of Health Sciences, Jagiellonian University Medical College, 31-126 Kraków, Poland; justyna.k.kot@uj.edu.pl (J.K.); dorota.matuszyk@uj.edu.pl (D.M.); 3 Faculty of Health Sciences, Vincent Pol University in Lublin, 20-853 Lublin, Poland; 4 Department of Obstetrics and Gynecology, Faculty of Health Sciences, Medical University of Lublin, Staszica 4-6 Street, 20-081 Lublin, Poland; natalia.wdowiak62@gmail.com (N.W.); melabojar@gmail.com (M.B.); wdowiakartur@gmail.com (A.W.); 5 Department of Obstetrics and Pathology of Pregnancy, Medical University of Lublin, Staszica 4-6 Street, 20-081 Lublin, Poland; michal.a.filip@gmail.com; 6 Second Department of Gynecology, Medical University of Lublin, Jaczewskiego 8, 20-090 Lublin, Poland; wrobelandrzej@yahoo.com; 7 Medical Faculty, Medical University of Lublin, 20-093 Lublin, Poland; wrobeljan@onet.eu; 8 Department of Cosmetology and Aestetic Medicine, Medical University of Lublin, 20-059 Lublin, Poland; jbartosinski@gmail.com

**Keywords:** COVID-19, infertility, quality of life, assisted reproductive technology (ART)

## Abstract

**Background/Objectives:** Infertile people experience a lot of psychological stress due to the inability to conceive and achieve pregnancy. Studies on the quality of life (QoL) of people undergoing infertility treatment typically show a lower QoL for couples struggling with reproductive problems. In recent years, a new factor that may have had a stressful impact on people treated for infertility is the COVID-19 pandemic. The aim of this study is to assess the impact of the COVID-19 pandemic on the QoL of Polish women treated for infertility and on the secretion of selected sex hormones. **Methods:** The study sample consisted of 600 women undergoing treatment due to infertility and 100 healthy women in a control group. The World Health Organization Quality of Life-BREF (WHOQOL-BREF) and the Polish version of the questionnaire Fertility Quality of Life (FertiQoL) were used for data collection. The levels of selected hormones were measured from blood samples. **Results:** The effects of the pandemic were visible primarily in the reduced QoL of patients. The QoL in terms of physical health, psychological health, social relationships, and environmental sphere was drastically reduced by COVID-19, especially among women treated with IVF (in vitro fertilization) and IUI (intrauterine insemination). The hormonal status of women treated for infertility during the pandemic significantly changed due to a decrease in FSH (follicle-stimulating hormone) and LH (luteinizing hormone) secretion and an increase in PRL (prolactin). **Conclusions:** The pandemic resulted in a decline in the QoL of women with reproductive problems. The quality of life was influenced by the type of therapy used during infertility treatment. The study also suggests a relationship between a decrease in the quality of life of persons treated for infertility during the pandemic and their hormonal status.

## 1. Introduction

Infertility is defined by the World Health Organization as the failure to achieve pregnancy after 12 months or more of regular unprotected sexual intercourse without using any contraceptives [1]. While classifying infertility, primary and secondary infertility may be distinguished. Primary infertility describes the situation in which the couple has never been able to conceive and achieve pregnancy, whereas secondary pregnancy is when pregnancy has been achieved in the past but there is a current problem with conceiving a baby [2].

According to estimations, at present, in Europe, the problem concerns approximately 20% of the population of reproductive age [3] and it constitutes a serious challenge in reproductive medicine and public health.

The causes of infertility should be sought both from the perspectives of men and women. In women, they include hormonal disorders, less often infections, adhesions in the minor pelvis, endometrial polyps, and chronic diseases, and abnormalities in the structure of reproductive organs are also important. The causes of infertility in men most often include abnormal semen parameters, testicular diseases, or past surgical procedures. When the etiology of reproductive disorders remains unexplained, infertility is considered as idiopathic [4]. Irrespective of the factor (male or female) in the case of diagnosing infertility, it should be remembered that the diagnosis is made in the context of a couple, and not in the context of an individual.

In the majority of cases, the diagnosis and treatment of infertility are not easy or quick processes, and they require patience, discipline, and regular medical visits. Long-term diagnostics may result in failure, causing frustration from both psychological and social aspects. A couple, especially for woman, may show social withdrawal caused by criticism of their environment (perceived as a conscious choice of a comfortable life) and the pressure from the immediate environment for them to fulfil their social role (of a parent) according to a schedule imposed by society. In such a situation, women most often avoid social contact and assume a feeling of guilt [5].

Studies on the quality of life of persons undergoing infertility treatment have been conducted for years by various researchers across the world. The results of these studies usually demonstrate a lower quality of life of couples struggling with reproductive problems, compared to those with confirmed fertility. The conditioning of this problem among Polish patients was investigated by the team of researchers conducting the presented study. The analyses performed show that the quality of life depends on reproductive problems, methods of infertility treatment, age, place of residence, and level of education. Extension of the duration of treatment exerted a negative effect on the quality of life. The quality of life of women undergoing infertility treatment differed according to their work pattern and whether they had children from a previous relationship [6]. However, these studies did not consider a new factor that might have affected the quality of life of patients: the COVID–19 pandemic.

The COVID–19 epidemic was first identified on 17 November 2019 in the city of Wuhan, in the Hubei province of central China, and on 11 March 2020 it was considered by the World Health Organization (WHO) as a pandemic [7]. The SARS–CoV–2 epidemic may have caused the occurrence of such symptoms as anxiety, depression, sleep problems, and additionally may have exerted an unfavourable effect on patients already diagnosed with psychological disorders, deepening their states of anxiety and depression [8]. These symptoms were primarily due to the imposed social isolation and the transmission of negative information through the media.

Considering the complex interactions between stress and the HPG (hypothalamo-pituitary–gonadal) axis, the connections between stress, stress relief methods, and successful conception in humans are poorly defined. Similarly, to date, cause and effect relationships between stress and infertility have not been successfully determined. It is well known that the HPG axis is regulated by peripheral and central mechanisms, and its effect on reproduction may depend on different types of stress, such as acute or chronic, and individual resilience. Stress exerts an effect on the hypothalamic GnRH (gonadotrophin-releasing hormone) pulse generator, reducing its ability to govern the pulsatile release of gonadotropins. Dysregulation in pulsatile GnRH secretion in the hypothalamus may account for hypothalamic amenorrhea and can lead to a wide range of reproductive disorders. Endocrine hormones in the hypothalamus regulate the secretion of pituitary prolactin, which diminishes the effect of stress and possibly, at least to some extent, reduces the responsivity to stress observed in pregnancy and during lactation, during which the levels of prolactin are elevated [9,10].

In association with the sudden occurrence of the pandemic, many couples undergoing treatment in infertility centres had to discontinue diagnostics or postpone their planned procedures. This generated costs (repeated examinations, subsequent visits, oocyte cryopreservation) and enhanced social frustration (extended time of trying to get pregnant, fear for own health and that of the baby). In Poland, during the pandemic, no lockdown was introduced in the centres dealing with infertility treatment, which enabled the continuation of studies concerning the quality of life of persons with reproductive problems. The aim of the presented study is to assess whether and to what extent the COVID–19 pandemic affected the quality of life of Polish women treated for infertility and their secretion of selected sex hormones.

## 2. Materials and Methods

### 2.1. Study Groups

A prospective cohort was examined in the OVUM medical centre in Lublin from December 2018 to December 2021. The criterion for inclusion into the study group (before the pandemic spanned from 2018 to 2019, during the pandemic from May 2020 to the end of 2021) was a diagnosis of infertility according to the WHO classification. Before the COVID pandemic, there were 300 women in the study group (100 treated without ART, 100 with IUI, and 100 with IVF). During the pandemic, the numbers in the individual groups were the same. The control group comprised women with confirmed fertility (before pandemic *n* = 50, during pandemic *n* = 50). The size of the groups was determined by the start of the pandemic. Women who declared undergoing psychiatric treatment or psychotherapy and those diagnosed with ovulation disorders according to WHO criteria were excluded from the study (Groups I, II, III). During the study, 18 questionnaires were incomplete and were rejected (7 from the study group before COVID and 8 during COVID, and in the control group, 1 and 2, respectively).

The recruitment process for both the study and control groups was conducted by means of two communication channels. The first channel employed the printing and distribution of advertisements concerning the study in local gynecological consultation rooms, while the second advertised the same content online. The advertisements were in Polish; therefore, the participants had to know the language on a communicative level. Participation in the study was voluntary. Consent for the study was obtained from the Bioethical Committee at the Medical University in Lublin, No. KE-0254/79/2017.

### 2.2. Description of the Research Tools

The study was conducted by applying the method of a diagnostic survey using standardized research tools: the World Health Organization Quality of Life-BREF (WHOQOL-BREF) [11] and the Polish version of the questionnaire Fertility Quality of Life (FertiQoL) [12].

The WHOQOL-BREF questionnaire is an abbreviated version of the WHOQOL-100, measuring the perception of the quality of life among both the healthy and the ill [13,14]. The WHOQOL-BREF consists of 26 items, the first two referring to general statements concerning subjective evaluation of the quality of life and the participant’s own state of health. The remaining 24 items concern four domains of functioning: physical health, psychological health, social relationships, and environmental health. Each of the 26 items is scored according to a 5-point scale; however, this differs by the terms used on the scale and the direction of replies, depending on the content of the question, which results from the construction of the questionnaire. The raw scores obtained from respondents on each scale should be converted into scores on a 0–100 scale in accordance with the WHO recommendations. A higher score obtained on the scale indicates a higher quality of life in a given domain.

In turn, the FertiQoL questionnaire is used for measuring the quality of life of persons with infertility problems. The FertiQoL consists of two parts, containing a total of 36 questions. In the FertiQoL questionnaire, a 5-point Likert scale is applied. Each item is assigned a value between 0 and 4, and higher scores on the scale indicate a better quality of life in relation to fertility. FertiQoL subscale scores are calculated and converted to scaled scores. Scaled scores are calculated to have a range from 0 to 100, which facilitates the comparison of scales. Most questions are scored in a positive direction. The results from reverse-scored questions are transformed accordingly. Similarly to the WHOQOL-BREF, the first two questions (A and B) help grasp an overall assessment of the state of physical health and satisfaction with the quality of life. However, the replies are not included in the overall score and only give additional information. The basic part of the questionnaire (CORE) contains 24 questions for the assessment of the quality of life of persons suffering from infertility in four dimensions: emotional, mind/body, relational, and social. The second part, TREATMENT, is an optional module containing 10 questions, and it consists of two subscales: treatment environment and tolerance to treatment. The overall result of the FertiQoL demonstrates the quality of life in two main domains.

Levels of FSH, LH, PRL, and AMH (anti-mullerian hormone) were measured on the third day of the cycle preceding ovulation. The levels of all hormones were determined in serum obtained from morning blood samples (5 mL). Levels of FSH, LH, TSH, and PRL were determined by an electrochemiluminescent method using a Cobas analyzer (Roche Diagnostics)—the reference values were FSH, 3.5–12.5 mIU/mL; LH, 2.4–12.6 mIU/mL; TSH, 0.2–4.5 μIU/L; and PRL, 4.79–23.3 ng/mL. The levels of AMH were determined by means of the AMH Gen II ELISA test (Beckman Coulter, Inc. United States) using an Euroimmun analyzer (reference range > 1.5 ng/mL).

### 2.3. Statistical Methods

Statistical analyses were conducted using STATISTICA version 13.3 software. Arithmetic mean (M) and standard deviation (SD) were estimated for continuous variables, and absolute numbers (*n*) and percentages (%) of the occurrence of items for categorical variables. The following statistical tests were used:

- Pearson’s chi-square test to compare the categorical characteristics between study groups;
- Student *t*-test to compare numerical characteristics, hormone concentrations, and quality of life between study groups;
- Pearson correlation coefficient to correlate BMI with hormone concentrations;
- F test analysis of variance to compare hormone concentrations between city, town, and rural area residents;
- Multiple regression to correlate total score of FertiQoL with characteristics and hormonal status in women treated for infertility.

The significance level was assumed to be 0.05.

## 3. Results

### 3.1. Characteristics of the Study Group

The analysis included data concerning 600 women undergoing treatment due to infertility, and 100 women in the control group.

Table 1 presents the characteristics of the study group. The mean age of the women in the group undergoing infertility treatment (group non-ART, group IUI, and group IVF) was higher during the period of the COVID–19 pandemic, compared to the period before the pandemic (*p* = 0.016), whereas in the control group, no statistical difference was observed. The majority of women in all groups in the study were urban inhabitants, had a higher education, and were characterized by a normal Body Mass Index (BMI). More than 76% of women treated for infertility had not yet had any children. The time over which the respondents had tried to have a child was 3.5 years on average, irrespective of the presence of the SARS–CoV–2 pandemic. Regarding occupational activity, the largest number of women performed non-physical work, and their net income per person in the household exceeded PLN 2000 monthly.

### 3.2. Levels of Hormones

In all groups in the study, a statistically significant increase in the level of PRL was observed, in addition to a decrease in FSH as well as in LH during the COVID–19 pandemic. The values of AMH and TSH did not significantly differ in individual groups during both periods analyzed (Table 2).

### 3.3. Quality of Life According to the WHOQOL–BREF Questionnaire

One of the main goals of the study was the comparison of the results obtained from the WHOQOL–BREF questionnaire (Table 3). In all groups in the study, the overall perception of the quality of life (Q1) was evaluated in lower terms during the COVID–19 pandemic. A statistically significant result was noted among women treated by the method of in vitro fertilization, and those not using assisted reproductive techniques (*p* < 0.001 each). In all groups receiving infertility treatment, statistically significant differences were found in the overall perception of health (Q2).

The quality of life in physical, psychological, social, and environmental domains was assessed in higher terms before the pandemic. The analysis showed significant results (*p* < 0.001) in all domains among women treated by the IUI method and in vitro fertilization. In the group of women treated using the non-ART method, no significant statistical differences were observed between the examined periods of time. In the control group, significant differences were noted only in the domain of physical health (*p* < 0.001).

### 3.4. Quality of Life According to the FertiQoL Questionnaire

A subsequent important aim of the study was the analysis of the individual domains included in the FertiQoL questionnaire, according to the duration of the COVID-19 pandemic (Table 4). The first two questions (A and B) present the self-reported state of health and the quality of life of respondents according to the FertiQoL questionnaire. In the case of IUI, the assessment of the state of health during the pandemic was slightly lower (lack of significant changes), and the evaluation of the quality of life also decreased (statistically significant differences, *p* < 0.001). Analysis of the differences in women treated by the non-ART methods did not show any differences in the assessment of the state of health and the quality of life both before or during the pandemic. Regarding the results of the IVF group, during the pandemic, a considerable deterioration was observed in the evaluation of health and the quality of life (*p* < 0.001).

The women treated for infertility generally assessed their quality of life in lower terms during the pandemic; however, not all components achieved statistically significant values. A decline in the results of total FertiQoL was the highest in women who received IVF treatment (54.7 vs. 38.4; *p* < 0.001). In the IUI group slightly better results were obtained and were also statistically significant (56.2 vs. 48.7; *p* < 0.001). A similar relationship was found regarding the Core FertiQoL. Analysis of changes in the treatment domain before and during the COVID-19 pandemic demonstrated statistically significant differences in women treated using the IVF method, compared to the non-ART and IUI groups. In women treated by non-ART methods, the components of quality of life according to the FertiQoL questionnaire did not significantly differ before and during the pandemic (*p* > 0.05 in all subscales).

### 3.5. Multivariable Regression Analysis

Table 5 presents the results of the multivariable regression analysis of total FertiQoL scores against the characteristics and hormonal status of women treated for infertility.

The women with a tertiary level of education had a significantly lower quality of life, compared to those with secondary level of education (*p* < 0.001). The women without children had a significantly lower quality of life, compared to those who had children (*p* = 0.001). The women who performed non-physical work had a significantly higher quality of life, compared to those engaged in mixed forms of work (both physical and non-physical) (*p* = 0.010).

The women with higher PRL and lower FSH levels had a significantly lower quality of life, compared to those with lower PRL and higher FSH levels (*p* = 0.47 and *p* = 0.039, respectively).

The quality of life among women treated for infertility did not correlate with age, place of residence, BMI, time trying to have a baby, income, LH, AMH, or TSH levels (*p* > 0.05).

### 3.6. Analysis of Hormonal Status

Table 6 illustrates the results of the analysis of hormonal status in the study group. No statistically significant correlations were found between hormone levels and BMI in the study group. No statistically significant relationships were found between hormone concentrations and the place of residence and education of women in the study group.

## 4. Discussion

Infertility is a very complex and multi-layered aspect which, according to the WHO, is classified into civilisation diseases. This problem results in a considerable deterioration in the quality of life, affecting psychical and psycho-social wellbeing [15]. Many studies have indicated a relationship between stress and infertility [16]. An additional burden of stress may be imposed by the long-term process of treatment, including the use of assisted reproductive techniques. A review by Gdańska et al. emphasizes the essence of the importance of anxiety and depression related to infertility and assisted reproductive techniques applied during treatment. In this context, studies clearly indicate a decrease in the quality of life among persons struggling with infertility [17].

Stress may exert an effect on human fertility in several ways. Firstly, it may disturb the psychological balance, leading to various problems in the sexual sphere, such as decreased libido. The activity of stress hormones has a direct effect on disorders in the functioning of the ovaries, delaying the maturation of follicles and ovulation, and also, to a considerable degree, on the process of spermatogenesis. From a biological perspective, stress causes the activation of body organs and systems. The response involves the general activation of the hypothalamic–pituitary–adrenal axis and sympathetic–spinal systems. From the point of view of fertility, the HPG is of a key importance—exposure to stress disrupts the pulsatile secretion of GnRH by the hypothalamus and limits the synthesis and release of gonadotropins, LH and FSH, from the anterior lobe of the pituitary gland. This, in turn, results in a decreased production of sex steroid hormones, including estrogen, progesterone, and testosterone. Thus, disorder in the functioning of the HPG axis has direct consequences for fertility in males and females [9,18].

During the period of study, an additional stressor was the outbreak of the SARS–CoV–2 pandemic, which, according to Bechmann et al., exerted an effect on all spheres of human life, including reproductive health. From this aspect, the researchers emphasized the consequences of psychological stress [18]. Persons struggling with the problem of infertility were painfully affected by the COVID–19 pandemic [19]. In many countries, lockdown was introduced, which forced couples to postpone their parenting plans [20,21]. In Poland, there was a possibility to continue therapy, which enabled this study concerning the assessment of the effect of the COVID–19 pandemic on the hormonal status and quality of life of women treated for infertility and allowed for the comparison of results with those from before the pandemic.

The presented results provide new information concerning the effect of the pandemic on the quality of life of women with reproductive problems. This study demonstrates that the overall quality of life of women treated for infertility was evaluated in lower terms during the COVID–19 pandemic, compared to the period before the pandemic. This was especially important in the case of treatment IVF and non-ART methods. Similar results were obtained regarding the overall perception of health in all groups treated for infertility. Women who received treatment due to infertility using IUI and IVF methods were more susceptible to the COVID–19 pandemic period and showed a stronger tendency towards a lower evaluation of individual domains of the quality of life, compared to the non-ART method and the control groups.

The scope of problems concerning the quality of life during the pandemic of coronavirus among patients treated for infertility is relatively limited. The results of a study by Gordon and Balsom demonstrated that the COVID–19 pandemic had a considerable effect on the decline in the overall quality of life and psychological health. More than a half of respondents confirmed a clinical level of the symptoms of depression. The researchers suggest that this was related to a delay or suspension of infertility treatment [22].

It is known that psychological and emotional health are the key elements that exert an effect on the quality of life. Data obtained from a systematic review by Irani et al. indicate that the majority of patients experienced negative emotions during the COVID–19 pandemic [23]. These results are also confirmed by a systematic review by Kirubarajan et al., suggesting that the COVID–19 pandemic caused unfavourable psychological effects in the sphere of reproductive health [24]. In turn, a study by Dong et al. showed a higher level of depression in women who experienced a delay in treatment, compared to those who gained access to therapy. In both cases, the researchers justified a higher frequency of depression occurrence with an increase in anxiety caused by the coronavirus pandemic, as well as by the suspension of treatment [25].

While analyzing the results of the presented study from the aspect of the quality of life of persons affected by infertility (FertiQoL), it was observed that women treated using ART evaluated their quality of life during the pandemic in lower terms. Similar results were obtained from the participants of a cross-sectional study by Biviá-Roig et al., carried out at three time points (before isolation, during isolation, and after resumption of reproductive treatment), indicating a higher level of anxiety and a lower quality of life during the period of isolation, compared to quarantine. In the above-mentioned study, the result of HADS showed significant differences between the three time points, with the lowest values noted during and after isolation, compared to the period before isolation. In turn, the result of the FertiQol–total demonstrated a downward tendency during isolation, which considerably decreased during the period after the resumption of reproductive treatment [26].

There are conflicting beliefs as to whether the decision to discontinue infertility treatment during the COVID–19 pandemic was justified. On the one hand, it was dictated by the need to ensure the safety of patients; however, on the other hand, studies show that the suspension or postponement of treatment had a negative effect on the psychological health of patients and their quality of life. Against this background, an interesting observation was presented by Qu et al., who compared the results of study participants during the outbreak of the pandemic and during the control period. The researchers found that due to the effective control of the COVID–19 pandemic, the psychological health of women treated using the ART method improved in all domains [27].

The hormonal status of women during the pandemic significantly changed as a result of a decrease in the secretion of pituitary gonadotropins and an increase in prolactin levels. Although these changes were statistically significant, from a clinical point of view, they should not considerably affect female fertility [10]. The effect of these changes should not be expected, especially for the results of treatment by the in vitro fertilization method, where the internal secretion of FSH and LH is inhibited and stimulation involves the administration of exogenous gonadotropins.

The observed changes in the secretion of FSH and LH may be explained by the effect of stress related to changes that occurred during the pandemic on the hypothalamic–pituitary–ovarian axis. The concept of stress has been controversial since the early part of the 20th century, when Cannon and Selye first identified the body’s physiological reaction to stress. Nevertheless, a stressor may generally be defined as a stimulus that disrupts body homeostasis, resulting in a response involving the activation of the hypothalamic–pituitary–adrenal (HPA) axis and the sympathomedullary pathway. Stressors can activate unique response patterns—signatures. In this context, stress is referred to as a stimulus, while the stress response is the reaction of the body to this stimulus. There are many types of stress which, according to the direct effect on the body, the endocrine, and the neural response, may be generally categorized into physical and psychological stress. Physical interoceptive, homeostatic, and systemic stressors relate to the direct disruption of tissue integrity, activation of the central amygdala and noradrenergic cells of the brainstem, and activation of the HPA axis and the sympathomedullary pathway. Psychological stressors— neurogenic, psychogenic, and emotional—relate to a threat to tissue impairment rather than immediate disruption, and to activation of the medial amygdala and neuronal activation in the caudal brainstem.

Recent studies conducted on rats suggest that prolactin promotes resilience to chronic stress. Rats which were more resilient to stress showed higher prolactin levels in their plasma [28]. This is in accordance with the results of the presented study regarding an increase in the PRL level in women during the COVID pandemic. Some studies demonstrated that psychological stress is related to low TSH levels [29,30]. There are reports that connect the secretion of thyroid hormones with the quality of life; however, these studies are not consistent [31]. The presented study did not confirm a relationship between the quality of life and TSH levels.

In the present study, we tried to evaluate which factors may have potential influence on the QoL of Polish women treated for infertility. As the results show, the overall scores on the FertiQoL did not correlate with age, place of residence, or income. One study showed a relationship and better results in the FertiQoL in respondents with a secondary level of education. In addition, the evaluations of the quality of life positively correlated with women who performed non-physical work. The results obtained in this respect are in contrast to other studies that suggest a higher quality of life in urban rather than rural areas and among older, better-educated respondents [6]. In turn, some studies in other countries have shown that the quality of life of younger women is better. In addition, with increasing age, hormonal balance changes and ovarian function gradually decreases, which results in a decrease in the success rate of IVF. This may further affect fertility pressure [32].

Undoubtedly, in the event of the pandemic or similar events that worsen the quality of life, patients undergoing infertility treatment should receive psychological support from professionals. Access to psychological help should be easy to implement. In the case of a lockdown, we recommend providing access to online support groups and adapting care to the virtual realm. At the same time, medical personnel treating infertility should be able to identify patients who require help. This can be achieved by introducing specialized surveys among patients, which can select patients who require support. Further research on this topic is very much needed.

It should be emphasized that this is one of the few studies investigating the pandemic’s impact on the quality of life of women treated for infertility. One of our strengths was the use of validated tools previously verified and used in many countries. Some limitations of this study should also be considered. The first limitation was the control group size. Expanding the sample size, particularly in the control group, could enhance the generalizability of the results. Additionally, the studies were conducted in a single centre and for a limited period over the COVID–19 pandemic. As a result of this limitation, further exploration of how the different stages of the pandemic (e.g., early phase, peak, and later phases) might have affected the results was not conducted. A phase-wise analysis could reveal more specific impacts on infertility treatment, as different stages may have influenced patients’ psychological wellbeing and the continuity of treatment in varying ways.

## 5. Conclusions

This study assessed the effect of COVID–19 on the sphere of reproductive health in women treated for infertility. The consequences were manifested primarily in a decrease in the quality of life of patients during the pandemic. The results of the functional variables obtained using two scales (WHOQOL–BREF and FertiQoL) were comparable. The levels of QoL within the scope of physical and psychological health, social relations, and the environmental sphere were drastically decreased by the COVID–19 pandemic, especially among women treated using IVF or IUI methods. A similar relationship was obtained in the fertility-specific domains of the quality of life: emotional, mind/body, relational, and social. In all the examined groups, the pandemic of SARS–CoV–2 exerted the smallest effect on the tolerance to treatment subscale. The presented study also suggests a relationship between a decrease in the quality of life of persons treated for infertility during the pandemic and their hormonal status.

## Figures and Tables

**Table 1:** Characteristics of the study group.

Variable, Parameter	IU or Category	Women Treated for Infertility			Control Group		
		Before COVID	During COVID	*p*	Before COVID	During COVID	*p*
		*n* = 300	*n* = 300		*n* = 50	*n* = 50	
Age, M ± SD	years	32.4 ± 4.5	33.3 ± 4.6	**0.016**	32.7 ± 3.7	34.2 ± 4.3	0.065
Place of residence, *n* (%)	city	130 (43.33)	132 (44.00)	0.986	27 (54.00)	24 (48.00)	0.672
	town	60 (20.00)	59 (19.67)		11 (22.00)	10 (20.00)	
	rural area	110 (36.67)	109 (36.33)		12 (24.00)	16 (32.00)	
Level of education, *n* (%)	secondary	68 (22.67)	69 (23.00)	0.923	10 (20.00)	12 (24.00)	0.629
	tertiary	232 (77.33)	231 (77.00)		40 (80.00)	38 (76.00)	
BMI, M ± SD	kg/m^2^	24.37 ± 4.70	24.44 ± 4.79	0.857	24.48 ± 5.08	25.89 ± 4.86	0.159
BMI, *n* (%)	underweight	21 (7.00)	20 (6.67)	0.991	2 (4.00)	3 (6.00)	0.957
	normal weight	171 (57.00)	172 (57.33)		28 (56.00)	27 (54.00)	
	overweight	70 (23.33)	68 (22.67)		10 (20.00)	11 (22.00)	
	obesity	38 (12.67)	40 (13.33)		10 (20.00)	9 (18.00)	
Have children, *n* (%)	yes, from the current relationship	33 (11.00)	34 (11.33)	0.965	n.a.	n.a.	n.a.
	yes, from a previous relationship	38 (12.67)	36 (12.00)		n.a.	n.a.	
	no	229 (76.33)	230 (76.67)		n.a.	n.a.	
Time trying to conceive a child, M ± SD	years	3.4 ± 2.2	3.6 ± 1.9	0.234	n.a.	n.a.	n.a.
Type of job, *n* (%)	manual	47 (15.67)	45 (15.00)	0.974	7 (14.00)	9 (18.00)	0.860
	non-manual	157 (52.33)	158 (52.67)		28 (56.00)	27 (54.00)	
	mixed	96 (32.00)	97 (32.33)		15 (30.00)	14 (28.00)	
Monthly net income per 1 person in a household (thousand PLN), *n* (%)	below 2	76 (25.33)	76 (25.33)	0.996	10 (20.00)	11 (22.00)	0.915
	2–2.5	119 (39.67)	120 (40.00)		20 (40.00)	18 (36.00)	
	above 2.5	105 (35.00)	104 (34.67)		20 (40.00)	21 (42.00)	

Student’s *t*–test or chi–square test was used. *n*, number of respondents; M, mean; SD, standard deviation; BMI, body mass index; PLN, Polish currency; n.a., not applicable. Bold *p*-values are those statistically significant (*p* < 0.05).

**Table 2:** Hormonal status in study group.

Hormone M ± SD	Women Treated for Infertility									Control Group		
	IUI			Non–ART			IVF					
	Before COVID N = 100	During COVID N = 100	*p*	Before COVID N = 100	During COVID N = 100	*p*	Before COVID N = 100	During COVID N = 100	*p*	Before COVID N = 50	During COVID N = 50	*p*
PRL (ng/mL)	14.9 ± 3.4	17.4 ± 3.6	**<0.001**	15.9 ± 3.5	20.0 ± 3.6	**<0.001**	14.9 ± 3.4	18.3 ± 3.6	**<0.001**	13.9 ± 3.4	16.3 ± 3.6	**0.001**
FSH (mIU/mL)	8.6 ± 1.1	5.3 ± 3.5	**<0.001**	8.5 ± 1.1	5.3 ± 3.4	**<0.001**	8.5 ± 1.1	5.3 ± 3.4	**<0.001**	9.0 ± 1.1	8.3 ± 2.4	0.203
LH (mIU/mL)	6.3 ± 0.9	4.3 ± 1.3	**<0.001**	6.2 ± 0.9	4.2 ± 1.2	**<0.001**	6.2 ± 0.9	4.1 ± 1.2	**<0.001**	6.9 ± 0.9	6.5 ± 1.2	0.076
AMH (ng/mL)	5.5 ± 0.6	5.6 ± 0.5	0.201	5.4 ± 0.6	5.5 ± 0.5	0.159	5.4 ± 0.5	5.5 ± 0.8	0.230	4.6 ± 0.8	4.7 ± 0.8	0.311
TSH (mIU/L)	1.9 ± 1.1	1.6 ± 1.1	0.056	1.5 ± 1.0	1.6 ± 1.1	0.414	1.5 ± 1.0	1.6 ± 1.1	0.506	1.8 ± 1.1	1.6 ± 1.1	0.352

Student’s *t*–test was used. N, number of respondents; M, mean; SD, standard deviation; non–ART, women treated for infertility without the use of assisted reproductive technology; IUI, intrauterine insemination; IVF, in vitro fertilization; PRL, prolactin; FSH, follicle–stimulating hormone; LH, luteinizing hormone; AMH, anti-mullerian hormone; TSH, thyroid stimulating hormone. Bold *p*-values are those statistically significant (*p* < 0.05).

**Table 3:** WHOQOL–BREF scores in the study groups.

Subscales of WHOQOL–BREF M ± SD	Women Treated for Infertility									Control Group		
	IUI			Non–ART			IVF					
	Before COVID N = 100	During COVID N = 100	*p*	Before COVID N = 100	During COVID N = 100	*p*	Before COVID N = 100	During COVID N = 100	*p*	Before COVID N = 50	During COVID N = 50	*p*
Q_1_: How would you rate your quality of life?	3.8 ± 0.6	3.7 ± 0.5	0.019	3.9 ± 0.7	3.6 ± 0.4	**<0.001**	4.1 ± 0.6	2.4 ± 0.7	**<0.001**	4.1 ± 0.5	3.9 ± 0.6	0.058
Q_2_: How satisfied are you with your health?	3.7 ± 0.5	3.5 ± 0.6	**0.023**	2.6 ± 0.5	2.3 ± 0.5	**<0.001**	4.0 ± 0.7	3.6 ± 0.5	**<0.001**	1.9 ± 0.7	1.9 ± 0.5	0.736
Physical health	60.8 ± 8.5	44.3 ± 5.5	**<0.001**	65.3 ± 8.3	63.6 ± 8.4	0.151	57.7 ± 7.5	35.5 ± 5.3	**<0.001**	72.0 ± 9.0	65.6 ± 8.7	**<0.001**
Psychological health	68.1 ± 9.3	40.3 ± 5.0	**<0.001**	71.7 ± 9.1	70.8 ± 9.9	0.514	66.3 ± 8.1	28.9 ± 6.1	**<0.001**	78.0 ± 10.2	76.4 ± 9.6	0.426
Social relationship	71.7 ± 12.5	44.5 ± 8.7	**<0.001**	72.9 ± 13.0	72.7 ± 13.1	0.911	72.3 ± 10.1	29.4 ± 8.5	**<0.001**	75.5 ± 12.8	74.7 ± 13.1	0.748
Environment	69.0 ± 8.3	44.4 ± 6.5	**<0.001**	70.3 ± 7.7	70.2 ± 7.6	0.864	69.1 ± 6.9	30.5 ± 5.1	**<0.001**	71.6 ± 9.3	69.6 ± 8.0	0.252

Student’s *t*–test was used. N, number of respondents; M, mean; SD, standard deviation; non–ART, women treated for infertility without the use of assisted reproductive technology; IUI, intrauterine insemination; IVF, in vitro fertilization; WHOQOL–BREF, Abbreviated World Health Organization Quality of Life Questionnaire. Bold *p*-values are those statistically significant (*p* < 0.05).

**Table 4:** FertiQoL scores in the women treated for infertility.

Subscales of FertiQoL M ± SD	Women Treated for Infertility								
	IUI			Non–ART			IVF		
	Before COVID N = 100	During COVID N = 100	*p*	Before COVID N = 100	During COVID N = 100	*p*	Before COVID N = 100	During COVID N = 100	*p*
How would you rate your health?	3.7 ± 0.6	3.6 ± 0.8	0.474	3.9 ± 0.6	3.7 ± 0.5	0.138	3.6 ± 0.7	2.2 ± 0.6	**<0.001**
Are you satisfied with your quality of life?	3.7 ± 0.5	3.2 ± 0.6	**<0.001**	3.8 ± 0.6	3.7 ± 0.5	0.162	3.5 ± 0.7	2.4 ± 0.8	**<0.001**
Total FertiQoL	56.2 ± 10.0	48.7 ± 6.7	**<0.001**	57.4 ± 11.4	56.6 ± 11.1	0.636	54.7 ± 9.8	38.4 ± 4.7	**<0.001**
Core FertiQoL	61.1 ± 13.3	47.6 ± 7.0	**<0.001**	63.2 ± 14.7	62.7 ± 14.3	0.778	60.2 ± 13.4	31.3 ± 12.5	**<0.001**
Emotional	51.2 ± 18.4	45.9 ± 13.4	**0.020**	54.1 ± 19.0	54.1 ± 18.6	0.976	54.1 ± 17.2	33.1 ± 6.9	**<0.001**
Mind/body	57.8 ± 17.8	42.9 ± 9.9	**<0.001**	60.0 ± 19.4	59.8 ± 18.8	0.918	57.7 ± 17.7	23.2 ± 6.6	**<0.001**
Relational	74.1 ± 16.3	53.3 ± 7.3	**<0.001**	76.1 ± 15.0	74.1 ± 13.5	0.318	70.1 ± 15.9	38.5 ± 8.0	**<0.001**
Social	61.1 ± 17.8	48.1 ± 9.3	**<0.001**	62.7 ± 17.5	60.5 ± 15.2	0.353	59.0 ± 17.2	30.4 ± 7.2	**<0.001**
Treatment FertiQoL	51.4 ± 9.6	49.8 ± 9.4	0.231	51.6 ± 11.1	50.6 ± 10.7	0.548	49.3 ± 8.9	45.6 ± 8.4	**0.003**
Environment	67.4 ± 14.3	64.2 ± 12.8	0.097	67.3 ± 15.8	65.7 ± 14.5	0.454	64.7 ± 12.6	57.3 ± 11.1	**<0.001**
Tolerability	35.4 ± 11.9	35.4 ± 11.9	0.980	35.8 ± 12.5	35.6 ± 12.4	0.886	35.4 ± 9.8	33.9 ± 10.9	0.295

Student’s *t*–test was used. N, number of respondents; M, mean; SD, standard deviation; non–ART, women treated for infertility without the use of assisted reproductive technology; IUI, intrauterine insemination; IVF, in vitro fertilization; FertiQoL, Fertility Quality of Life tool. Bold *p*-values are those statistically significant (*p* < 0.05).

**Table 5:** Multivariable regression analysis results of total FertiQoL scores against characteristics and hormonal status in women treated for infertility (N = 600).

Covariate	IU or Category	b	*p*
Age	years	−0.13	0.225
Place of residence	city	1.19	0.070
	town	0.72	0.357
	rural area	reference	
Level of education	secondary	reference	
	tertiary	−2.41	**<0.001**
BMI	kg/m^2^	0.10	0.316
Having children	yes	reference	
	no	−1.39	**0.010**
Time trying for a baby	years	−0.32	0.153
Type of job	manual	−0.63	0.495
	non-manual	1.74	**0.010**
	mixed	reference	
Monthly net income per 1 person in a household (thousand PLN)	below 2	−0.83	0.263
	2–2.5	−0.15	0.814
	above 2.5	reference	
PRL	ng/mL	−0.15	**0.047**
FSH	mIU/mL	0.08	**0.039**
LH	mIU/mL	0.02	0.528
AMH	ng/mL	−0.03	0.347
TSH	mIU/L	0.01	0.815

General linear regression was used. b—regression slope term, i.e., mean change in total FertiQoL scores per unit of a covariate; BMI, Body Mass Index; PLN, Polish currency; PRL, prolactin; FSH, follicle–stimulating hormone; LH, luteinizing hormone; AMH, anti-mullerian hormone; TSH, thyroid stimulating hormone. Bold *p*-values are those statistically significant (*p* < 0.05).

**Table 6:** Analysis of hormonal status against body mass index (BMI) and characteristics in women treated for infertility (N = 600).

COVID	Unit or Category	Parameter	PRL (ng/mL)	FSH (mIU/mL)	LH (mIU/mL)	AMH (ng/mL)	TSH (mIU/L)
before	BMI (kg/m^2^)	r	0.047	0.036	−0.018	−0.022	−0.041
		*p*	0.417	0.535	0.756	0.704	0.479
during		r	0.021	−0.060	−0.048	−0.041	−0.094
		*p*	0.717	0.300	0.407	0.479	0.104
before	city	M ± SD	14.7 ± 0.4	8.7 ± 2.1	6.0 ± 0.6	5.1 ± 0.9	1.8 ± 1.1
	town	M ± SD	14.0 ± 2.4	8.5 ± 1.8	5.9 ± 0.7	4.5 ± 0.8	1.9 ± 1.8
	rural area	M ± SD	14.9 ± 8.4	8.0 ± 1.9	6.9 ± 0.9	5.9 ± 0.8	1.7 ± 1.0
	*p*		0.198	0.236	0.187	0.435	0.231
during	city	M ± SD	17.1 ± 3.9	5.0 ± 3.4	4.0 ± 1.3	5.6 ± 0.5	1.6 ± 1.1
	town	M ± SD	16.9 ± 3.9	5.1 ± 3.9	4.3 ± 1.5	5.0 ± 0.8	1.3 ± 1.2
	rural area	M ± SD	20.0 ± 2.1	5.2 ± 3.3	4.1 ± 1.8	5.1 ± 0.9	1.7 ± 1.1
	*p*		0.098	0.785	0.371	0.531	0.385
before	secondary	M ± SD	14.0 ± 1.8	8.8 ± 2.2	6.1 ± 1.5	5.0 ± 1.8	1.7 ± 1.2
	tertiary	M ± SD	14.5 ± 2.3	8.4 ± 1.9	5.8 ± 1.6	4.6 ± 1.7	1.9 ± 1.3
	*p*		0.100	0.142	0.169	0.093	0.257
during	secondary	M ± SD	17.0 ± 3.8	5.1 ± 3.6	4.1 ± 1.4	5.5 ± 1.6	1.4 ± 1.2
	tertiary	M ± SD	16.7 ± 3.5	5.2 ± 3.7	4.2 ± 1.6	5.1 ± 1.5	1.2 ± 1.1
	*p*		0.541	0.843	0.640	0.057	0.196

Pearson correlation coefficient and F test analysis of variance were used. M, mean; SD, standard deviation; r, Pearson correlation coefficient; BMI, Body Mass Index.

## Data Availability

The datasets generated during and/or analyzed during the current study are available from the corresponding author on reasonable request.
